# Anthropometric profiles and body composition of male runners at different distances

**DOI:** 10.1038/s41598-023-45064-9

**Published:** 2023-10-25

**Authors:** Aleksandra Stachoń, Jadwiga Pietraszewska, Anna Burdukiewicz

**Affiliations:** Faculty of Physical Education and Sport Sciences, Wroclaw University of Health and Sport Sciences, al. Ignacego Jana Paderewskiego 35, 51-612 Wrocław, Poland

**Keywords:** Anatomy, Health care

## Abstract

Anthropometric parameters are crucial prerequisite to achieve success in professional running sports. However, it is not clear how these parameters are relevant for athletes performing on a less demanding sport level as academic competitions. To help coaches and selectors working on this level, we have explored anthropometric variables and body composition in 68 academic athletes: 26 sprinters, 22 middle distance runners, and 20 long distance runners. Sprinters have a more massive body shape, shorter lower legs in relation to the length of the thigh, broader shoulders and narrower hips, greater musculature and cellular mass. A slender figure, a longer shin, and the greatest subcutaneous fat and extracellular mass characterize long-distance runners. Middle-distance runners are the slimmest, and have a narrow trunk and little subcutaneous fat. Sprinters and long-distance runners are mesomorphic, while middle-distance runners present more mixed mesomorph-ectomorph type. The principal component analysis highlighted the importance of the overall size of the body, limbs musculature and the length of the lower limb together with its segments, and also body fatness. This approach emphasized the morphological distinctiveness of runners at particular distances and allows the use of somatic features as predictors of running performance.

## Introduction

Running is the most popular and, at the same time, the simplest form of movement that brings multi-directional benefits to the body, including improving the functioning of the heart, nervous and digestive systems^1^. The popularization of this sport among amateurs is facilitated by the fact that it can be practiced at various distances, and various technical solutions are available to support training control^2,3^. In recent years, the popularization of running as a form of physical recreation has resulted in this issue being reflected in numerous scientific works^4^. Physiological aspects of running performance were studied, and issues related to running economy and energy costs were analyzed^5,6^. In addition, biomechanical aspects affecting the running economy have been studied^7–9^.

The issue of morphological diversity of runners focused on the characteristics of body proportions, body composition and somatic structure of competitors of specific distances and the impact of anthropometric features on the results achieved by athletes^10^. It has been shown that professional athletes (Olympic champions, finalists, and running event participants) are clearly differentiated in age, height and body weight^11,12^. Sprint champions tend to be heavier than lower-ranked competitors, while distance runners show the opposite trend^11,13^. A cohort study including elite and leisure runners shows that body composition is a better predictor of running performance than body mass index. Furthermore, fat mass was found to be negatively associated with running speed. High values of the fat-free index had a positive effect on the performance of women, while no such relationship was found for men^14^. In turn body composition studies of University level male track and field athlete of India have shown that sprinters have the lowest body fat. However, there was no significant difference between middle and long distance runners^15^. The Sheldon’ typology modified by Heath and Carter^16^ is often used to assess the physique in sports. That typology allows the assessment of body shape in the form of a somatotype that is understood as a description of the current morphological state of an individual. The somatotype is expressed by three numbers, each of which represents one of the basic components: endomorphy, mesomorphy and ectomorphy^17^. Endomorphy is relative fatness, mesomorphy is characterized by relative musculo-skeletal robustness and ectomorphy is relative linearity or slenderness of a physique. Highly trained athletes also differ from the general population of athletes by having less endomorphy and greater mesomorphy. Long distance runners who do mostly aerobic training are less endomorphic and mesomorphic but have more ectomorphy than other athletes^16^. Similar trends have been reported in the young elite middle and long distance runners^18^ and Iranian cross-country runners^19^. However, the cited studies had limitations related to, e.g., the number of studied athletes and their sports level.

As mentioned above, the body morphology of athletes is the result of selection and adaptation of the body to training loads, which differ in individual disciplines^20^. Running at different distances makes it necessary to use various training methods^21^. The volume and intensity of training are adapted to the distance the athlete will face. Optimizing selection processes and training methods based on the somatic factors seem to be essential to success in running at particular distances^22^. Our study aims to determine anthropometric profiles, including body size and proportions, somatotype and body composition of long-, middle- and short-distance runners. Our hypothesis is: the long-, middle- and short-distance runners are diversified in body size and proportions, somatotype as well as in body composition, which may be called as ‘various anthropometric profiles’.

## Materials and methods

### Participants

Sixty eight male college athletes (age 20.7 ± 2.05 yrs old) participated in the study. This sample included 26 sprinters (S), 22 middle distance runners (M) and 20 long distance runners (L). The runners were classified into the S, M or L groups according to their declaration of participation in sprints (200 m and 400 m), middle distances (800 m and 1500 m) and long distances (3000 m, 5000 m, 10,000 m). The athletes were involved in regional and national level competitions and trained at least 4 times a week for 2 h per day. Age and training experience did not significantly differ among the surveyed athletes: sprinters (20.37 ± 1.71 yrs old, 5.16 ± 2.21 years of experience), middle-distance runners (20.31 ± 1.55 yrs old, 5.50 ± 2.46 years of experience), long-distance runners (21.39 ± 2.74 yrs old, 6.75 ± 2.34 years of experience).

Academic athletes practice sports in the clubs of the Academic Sports Associations functioning at Wrocław universities. The athletes who fulfilled these criteria inquired for the study. The criterion for inclusion in the study was at least a 3 years of practice and competed at national level, no injuries and no special diets in the period preceding the study. The conditions for exclusion were a break in training and injuries or diseases that prevented the measurements.

The research was approved by the Senate’s Research Bioethics Commission of the Wroclaw University of Health and Sport Sciences, Poland [consent number 2/2020], and conducted according to the requirements stipulated in the Declaration of Helsinki. Participants were fully informed about all experimental procedures and written informed consent was obtained from all of them.

### Measurements and calculations

The measurements were carried out taking into account the training periodization, at the beginning of the preparation period and in the Central Research Laboratory of Wroclaw University of Health and Sport Sciences, Poland (Quality Management System Certificate: PN-EN ISO 9001:2015—Certificate Reg. No.: PW-15105-22X). All participants visited the laboratory once and underwent a series of measurements.

Measurements were made by experienced anthropometrists in the morning at room temperature about 22-24ºC. Anthropometric measurements were performed following measurement protocols established by the International Society for the Advancement of Kinanthropometry (ISAK). Each anthropometrist took a series of measurements assigned to them and was accompanied by a person recording them. Measurements were taken on the right side of the participant’s body. Anthropometric equipment from GPM Siber Hegner Machinery Ltd. was used. (Zurich, Switzerland): an anthropometer, a sliding caliper—Martin type, a spreading caliper, a skinfold caliper, an anthropometric tape. Body weight was measured using an electronic scale with an accuracy of 0.1 kg (Fawag, Lublin, Poland). Each measure was taken two times by the same investigator. Technical error of measurement was < 3% for skinfolds, and < 1% for breadths, lengths and girths. The mean values were used in the statistical analysis.

The results of measurements recommended for monitoring athletes^23^ were included in the study. Heights, lengths, widths and circumferences were measured to the nearest 0.1 cm: body height, lower limb height to *trochanterion* point, thigh length between *trochanterion* and *tibiale laterale* points, tibia length between *tibiale mediale* and *sphyrion tibiale* points, foot length, upper limb length between *acromiale* and *dactylion* points, arm length between *acromiale* and *radiale points*, forearm length between *radiale* and *stylion points*, biacromial breadth, biiliocristal breadth, biepicondylar humerus breadth, biepicondylar femur breadth, ankle breadth between *malleolare tibiale* and *malleolare fibulare* points. The following girths were measured: chest at the level of the *mesosternale* point, gluteal at the level of the greatest posterior protuberance of the buttocks, arm flexed, forearm, thigh taken 1 cm below the level of the gluteal fold and calf. Skinfold sites were landmarked at the subscapular, abdominal, supraspinale, triceps, forearm, front thigh and medial calf. All sites were then measured using caliper with 10 g × mm^−2^ constant pressure.

The measured features were used to calculate the following indices: body mass index BMI (body mass/body height^2^ [kg/m^2^]), lower limb length index (lower limb length/body height), upper limb index (upper limb length/body height), crural index (tibia length/thigh length), biacromial index (biacromial breadth/body height), biiliocristal index (biiliocristal breadth/body height), biiliocristal-acromiale index (biiliocristal breadth/biacromial breadth), bone massiveness index (biepicondylar femur breadth + ankle breadth/body height), flexed arm girth index (flexed arm circumference/arm length), forearm girth index (forearm circumference/forearm length), thigh girth index (thigh circumference/thigh length), calf girth index (calf circumference/tibia length).

In addition, two indices characterizing subcutaneous fat were calculated: fat distribution index and subcutaneous fat index. The fat distribution index is the quotient of the sum of skinfolds on limb segments (∑ triceps + forearm + front thigh + medial calf) and trunk (∑ subscapular + supraspinale + abdominal). Subcutaneous fat index (SFI) takes into account trunk and extremity skinfolds and body height (∑ trunk skinfolds + ∑ limbs skinfolds/body height)^24^.

The somatotype of each subject was also determined according to Sheldon’s method, modified by Heath and Carter^16^. Somatotype Calculation and Analysis software classified the average somatotype of each group and illustrated the outcome in a somatotype chart^25^.

The non-invasive bioelectrical impedance method assessed body composition with tetrapolar version hand-to-foot electrodes (BIA 101 analyzer, Akern, Bodygram 1.31 software). Measurements were made considering the manufacturer’s rules for obtaining correct results. The following features were used in the analysis: fat mass (FM) [kg, %], body cell mass (BCM) [kg, %] and extracellular mass (ECM) [kg, %].

An interview was conducted with the respondents to collect information on the date of birth and training experience, diets and supplements, and the occurrence of injuries.

### Statistical analysis

Statistical analysis was performed using the Statistica 13.3 package (TIBCO Software Inc.). Descriptive statistics were used for the quantitative analysis of the collected data. The Shapiro–Wilk test was used to examine the distributions of the analyzed features. Differentiation in the level of development of the analyzed features between the groups was assessed using the analysis of variance and post-hoc Tukey HSD test for unequal samples. The results in the text and tables are presented in the form of mean and standard deviation. The significance level for all tests and statistical procedures was set at an p value of 0.05.

Differences in somatic composition were examined using the SANOVA—Somatotype Analysis of Variance procedure^25^. A ternary plot was used to examine the relationship between the three components of body composition (FM, BCM, ECM) in groups of runners. The distribution of individual competitors’ points in the three body composition variables system was assessed using the χ^2^ test. Principal component (PC) analysis was performed, expressing a linear combination of the morphological variables. The analysis was preceded by a Box-Cox transformation, which enabled the principal components to be based on correlations. The number of factors was defined using the Kaiser criterion^26^.

### Institutional review board

The study was conducted according to the guidelines of the Declaration of Helsinki and approved by the Ethics Committee of the University School of Physical Education in Wrocław, Poland (2/2020).

### Informed consent

Written informed consent has been obtained from the patient(s) to publish this paper.

## Results

Short-distance runners are characterized by significantly higher body weight than competitors from other groups (Table 1). Body height, length of the lower and upper limbs as well as their segments did not show any significant differences between the groups of runners. Among the width features, only the biacromial diameter is significantly wider in the group of sprinters compared to middle-distance runners. The massiveness of the skeleton assessed by the width of the elbow, knee and inner ankle reaches the highest values among examined sprinters. All the analyzed circumferences of the trunk and limb segments are the largest in the group of short-distance runners. The significantly thickest supraspinale and triceps skinfolds are characteristic of long-distance runners. Also, the sum of skinfolds on the trunk and limb segments is significantly greater in long-distance runners.

**Table 1:** Statistical characteristics and inter-group differences of the anthropometric features in short (S), middle (M) and long (L) distance runners (SD—standard deviation; ^a^significantly different from M; ^b^significantly different from L).

Group of runners Variable	S	M	L	*p*
	Mean (SD)	Mean (SD)	Mean (SD)	
Body mass [kg]	74.3 (6.56)^b^	69.2 (7.77)	67.6 (8.99)	0.011
Body height [cm]	180.6 (6.16)	181.3 (6.33)	177.3 (6.91)	0.109
Lower limb length [cm]	95.3 (3.99)	95.9 (4.34)	94.0 (3.89)	0.297
Thigh length [cm]	47.6 (2.17)	47.3 (2.22)	46.7 (2.07)	0.183
Tibia length [cm]	40.2 (1.89)	40.6 (2.13)	40.2 (1.77)	0.219
Foot length [cm]	27.0 (1.35)	26.7 (1.41)	26.6 (1.30)	0.603
Upper limb length [cm]	78.7 (3.02)	79.1 (3.21)	78.1 (3.13)	0.598
Arm length [cm]	33.8 (1.62)	34.0 (1.42)	33.6 (1.44)	0.695
Forearm length [cm]	26.1 (1.13)	26.4 (1.49)	25.8 (1.32)	0.259
Biacromial breadth [cm]	41.5 (1.86)^a^	40.0 (1.72)	40.7 (2.05)	0.024
Biiliocristal breadth [cm]	28.0 (1.50)	27.3 (1.55)	28.2 (1.92)	0.206
Humerus breadth [cm]	7.1 (0.48)	6.9 (0.37)	7.0 (0.67)	0.361
Femur breadth [cm]	9.8 (0.44)	9.6 (0.36)	9.8 (0.52)	0.185
Ankle breadth [cm]	7.7 (0.37)	7.4 (0.42)	7.5 (0.40)	0.130
Chest girth [cm]	95.3 (3.88)	93.2 (4.41)	93.6 (6.64)	0.333
Gluteal girth [cm]	96.2 (3.83)	94.5 (4.48)	93.6 (4.15)	0.111
Arm flexed girth [cm]	31.2 (1.75)^ab^	28.9 (1.63)	29.4 (2.37)	0.000
Forearm girth [cm]	25.9 (1.71)	24.8 (1.53)	25.1(1.55)	0.058
Thigh girth [cm]	55.5 (3.01)^ab^	53.1 (3.10)	51.8 (3.14)	0.001
Calf girth [cm]	37.1 (2.46)^a^	35.3 (2.65)	35.5 (2.28)	0.022
Subscapular skinfold [mm]	7.8(1.32)	7.1 (1.10)	8.1 (2.19)	0.095
Supraspinale skinfold [mm]	5.7 (1.44)^b^	5.6 (1.51)^b^	7.6 (3.47)	0.009
Abdominal skinfold [mm]	6.1(1.75)	6.2 (1.88)	7.2 (2.88)	0.195
Triceps skinfold [mm]	4.4 (1.36)^b^	4.6 (1.28)^b^	6.0 (2.56)	0.007
Forearm skinfold [mm]	3.1 (0.59)	3.0 (0.38)	3.3 (0.65)	0.119
Front thigh skinfold [mm]	7.9 (1.06)	7.4 (1.12)	8.2 (1.51)	0.107
Medial calf skinfold [mm]	3.8 (0.83)	3.7 (0.97)	4.4 (1.23)	0.101
∑ trunk skinfolds	19.6 (3.57	18.9 (3.76)^b^	22.9 (7.87)	0.038
∑ limbs skinfolds	17.2 (3.32)^b^	17.0 (3.21)^b^	20.3 (5.11)	0.010

BMI is significantly higher in the group of sprinters. The smallest massiveness is characteristic of middle-distance runners (Table 2). The general proportions of the length of the upper and lower limbs are similar in all groups of runners, but the proportions of functional segments differ. Sprinters are characterized by a significantly shorter tibia in relation to the length of the thigh compared to other groups of runners. They also have broader shoulders in relation to body height. The highest values of the hip width index characterize long-distance runners. The lowest values of the discussed indicator occurred among sprinters. The relative massiveness of the epiphysis of the lower limb is significantly lower among short- and medium-distance runners. Significantly larger girths of limb segments in relation to their length occurred among sprinters compared to other groups.

**Table 2:** Statistical characteristics and inter-group differences of the anthropometric indices, somatotype’s components and body composition in short (S), middle (M) and long (L) distance runners (SD—standard deviation; ^a^significantly different from M; ^b^significantly different from L).

Group	S	M	L	*p*
	Mean (SD)	Mean (SD)	Mean (SD)	
Body proportions				
Body mass index	22.76 (1.42)^ab^	20.99 (1.54)	21.44 (1.89)	0.001
Lower limb length index	52.75 (1.31)	52.88 (1.17)	53.00 (1.37)	0.801
Upper limb length index	43.59 (1.02)	43.61 (0.91)	44.06 (1.12)	0.244
Crural index	84.52 (2.12)^ab^	85.91 (1.89)	86.61 (1.90)	0.002
Biacromial index	22.98 (1.05)^a^	22.05 (1.02)^b^	22.97 (1.20)	0.007
Biiliocristal index	15.50 (0.90)	15.06 (0.79)^b^	15.87 (0.86)	0.012
Biiliocristal-acromiale index	67.48 (3.34)	68.37 (3.95)	69.19 (3.71)	0.294
Bone massiveness index	9.66 (0.39)^a^	9.37 (0.20)^b^	9.75 (0.47)	0.002
Flexed arm girth index	92.69 (6.94)^ab^	85.14 (5.14)	87.69 (6.97)	0.000
Forearm girth index	99.33 (6.62)^a^	94.13 (7.40)	97.71 (6.75)	0.038
Thigh girth index	116.62 (7.04)^b^	112.44 (6.67)	111.57 (6.26)	0.026
Calf girth index	92.34 (6.67)^a^	86.02 (6.01)	87.96 (5.74)	0.002
Fat distribution index	0.89 (0.18)	0.91 (0.18)	0.92 (0.20)	0.823
Subcutaneous fat index	20.44 (3.52)^b^	19.81 (3.25)^b^	24.33 (6.23)	0.003
Somatotype components				
Endomorphy	1.68 (0.38)^b^	1.60 (0.31)^b^	2.11 (0.76)	0.004
Mesomorphy	4.94 (1.00)^a^	3.82 (0.75)^b^	4.72 (1.12)	0.000
Ectomorphy	2.90 (0.77)^a^	3.81 (0.80)	3.36 (0.84)	0.001
Body composition				
% Fat mass	17.06 (3.31)	18.71 (3.57)	18.91 (2.52)	0.095
% Extracellular mass	31.53 (7.27)	32.89 (5.82)	33.22 (3.56)	0.583
% Body cell mass	51.34 (6.56)^ab^	48.40 (7.45)	48.03 (3.13)	0.035

The distribution of subcutaneous fat on the limbs and trunk expressed by the fat distribution index does not significantly differ between the groups of runners (Table 2). However, among the study participants, there was a tendency to increase the fatness of the limbs in relation to the trunk with the lengthening of the distance. The lowest adiposity of the limbs in relation to the adiposity of the trunk occurs in sprinters, while the highest—applies to the group of long-distance runners. The content of subcutaneous fat in relation to body height, expressed by the subcutaneous fat index, is significantly lower in the groups of short- and medium-distance runners.

**Figure 1:**
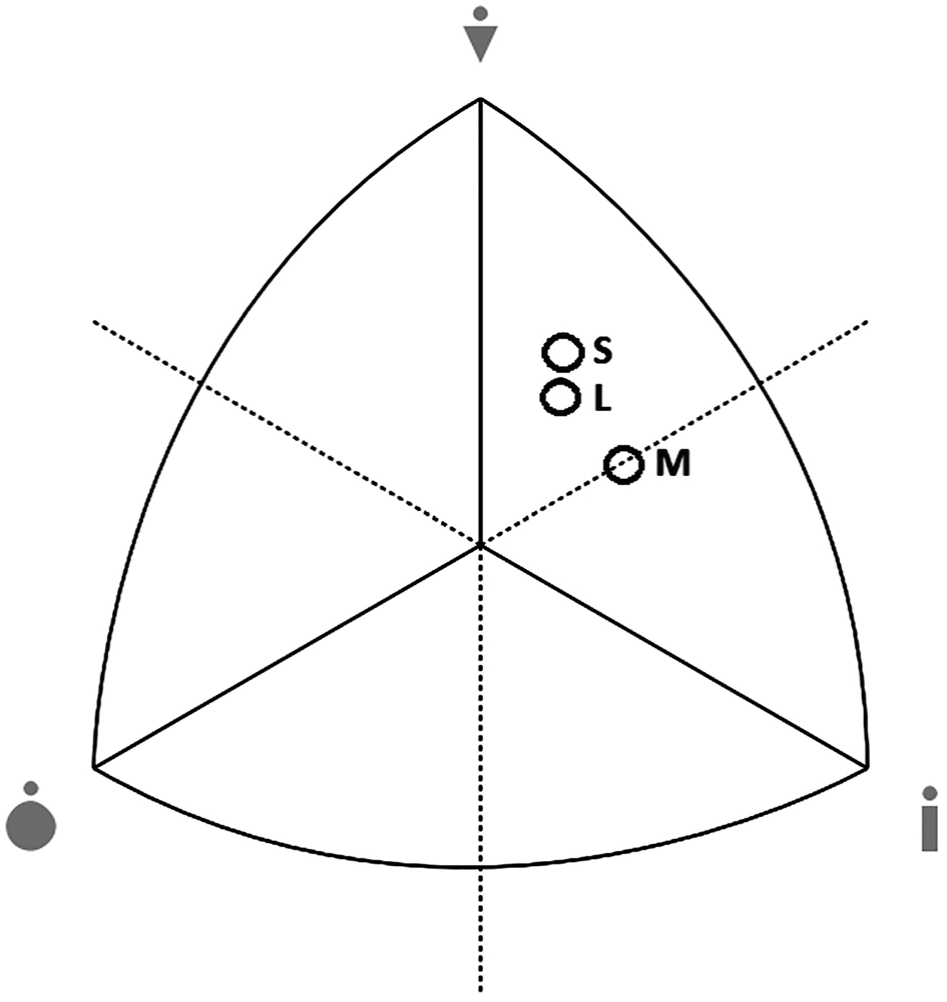
Mean of somatotypes in groups of male runners (S—short distance, M—middle distance, L—long distance).

Endomorphy is significantly lower in groups of short and middle distance runners than in long distance runners (Table 2). On the other hand, the mesomorphic component reaches significantly higher values in the group of short and long distance runners. However, significantly greater ectomorphy is characteristic of middle distance runners compared to sprinters. The average somatotype of middle runners are mesomorph-ectomorph (1.60–3.82–3.81), while sprinters (1.68–4.94–2.90) and long distance runners (2.11–4.72–3.36) are ectomorphic mesomorphs (Fig. 1). The somatotype variance analysis showed a statistically significant difference in the somatotypes of runners (F = 7.41; *p* = 0.001).

**Figure 2:**
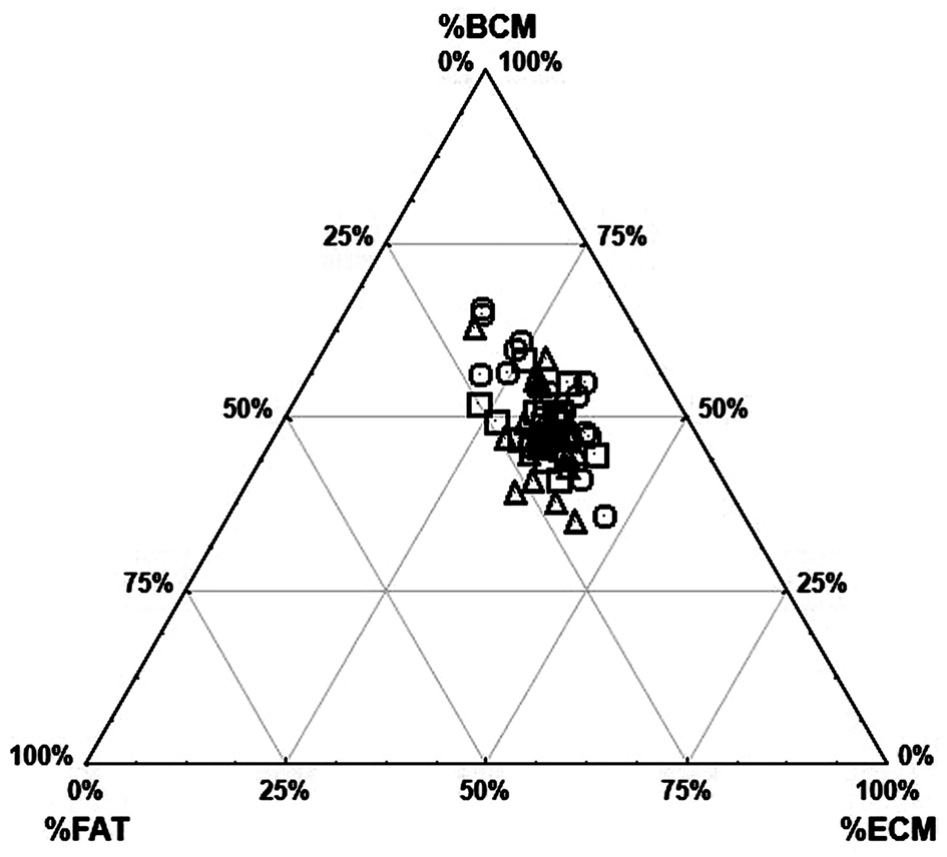
Distribution of runners in the system of three variables of body composition (○—short distance, Δ—middle distance, □—long distance).

The average values of the fat and extracellular mass percentages do not show statistically significant differences between the groups. In contrast, cell mass is significantly higher in sprinters compared to other groups. Distributions of individual competitors’ points in the system of three body composition variables (Fig. 2) assessed with the χ^2^ test also show statistically significant differences between groups (χ^2^ = 31.49, *p* < 0.05).

As a result of the principal components analysis, three principal components were identified, explaining the problem in approximately 74% (Table 3). The first principal component (PC1), which has the greatest part in total variability, is highly correlated with body weight and height, shoulder width and hip width, length of the lower limb and its segments, length of the upper limb, as well as with the muscle circumferences of the arm, forearm, thighs and thighs. Thus, the mentioned variable characterizes the overall size of the body. The second principal component (PC2) is positively correlated with muscle circumferences and negatively correlated with the length of the lower limb, the length of the thigh and the tibia. It can therefore be concluded that PC2 characterizes the massiveness and proportions of the limbs. This principal component divides the participants into two groups: (1) athletes with well-developed musculature, especially of the upper limb, and (2) athletes with a tendency to have longer lower limbs. In the case of the third principal component (PC3), the most diagnostic features are the sum of skinfolds on the trunk and the sum of skinfolds on the limbs. This component characterizes body fatness.

**Table 3:** Principal components loadings and correlations between the components and original variables.

Variable	PC1	PC2	PC3
Eigenvalues	7.02	2.52	1.59
% total variance	46.82	16.77	10.63
Cumulative eigenvalues	7.02	9.54	11.13
% cumulative	46.82	63.59	74.21
Factor loadings			
Body mass	0.91	0.27	0.05
Body height	0.84	− 0.35	0.03
Leg length	0.84	− 0.49	− 0.04
Thigh length	0.82	− 0.43	− 0.03
Tibia length	0.73	− 0.54	0.02
Foot length	0.77	− 0.13	0.14
Arm length	0.79	− 0.39	0.06
Biacromial breadth	0.54	0.35	− 0.05
Biiliocristal breadth	0.65	0.06	0.06
Arm flexed girth [cm]	0.66	0.66	− 0.18
Forearm girth [cm]	0.64	0.64	− 0.08
Thigh girth [cm]	0.73	0.44	− 0.10
Calf girth [cm]	0.65	0.42	− 0.23
∑ trunk skinfolds	0.17	0.30	0.81
∑ extremities skinfolds	0.03	0.11	0.90

Table 4 summarizes the mean principal component scores of the athletes from different distances. It can be concluded that the PC2 and PC3 components are significantly differentiated. The sprinter group’s overall body size (including massiveness and muscularity) is slightly higher. The second principal component PC2 substantially differs between the studied groups. Intergroup differentiation in the light of PC3 is also statistically significant. There is clearly a higher level of development of the analyzed muscle girths in short-distance athletes. Attention is drawn to the distinctiveness of long-distance runners, characterized by greater subcutaneous fat compared to competitors from other groups.

**Table 4:** Mean principal component scores of the runners from different groups (S—short distance; M—middle distance; L—long distance; ^a^significantly different from M; ^b^significantly different from L).

Group	S	M	L	*p*
PC1	0.86	− 0.23	− 0.69	0.100
PC2	0.74^a^	− 0.91	0.03	0.001
PC3	− 0.41^b^	− 0.19	0.60	0.010

## Discussion

In accordance with the reviewer's comment, we have introduced in the manuscript the following sentence: The results obtained make it possible to present the detailed somatic characteristics of runners at different distances, confirming the hypothesis that their anthropometric profiles are differentiated. The surveyed men were of similar age and had similar training experience. It is well known that running performance is determined by power output and running efficiency^6^. The first factor is related to the athlete’s physiological profile, while the second factor characterizes the efficiency in the conversion of power to translocation. It is directly related to the athlete’s biomechanical profile and depends on anthropometric dimensions, limb morphology, and learned and developed movement patterns^7,9^.

As shown in other studies, height and length dimensions were slightly larger in the group of middle-distance runners^27^. The body height of the athletes from the study groups was similar and oscillated around the value of the 50th percentile (178.7 cm) for the nationwide population^28^. This suggests that this parameter is not important in the selection process for specific running distances. Body shape assessed by BMI turned out to be slimmer in the middle- and long-distance runners groups than in sprinters, which was also indicated by other researchers^29^. In previously published studies, differences in body weight were noted depending on the results obtained by runners. The medalists of the Olympic Games in the distances of 100, 200, and 400 m were heavier compared to the finalists and other participants. In turn, the medalists at distances of 5,000 m and 10,000 m and the marathon were lighter than the finalists and other competitors participating in these distances^11^. High body weight and BMI values in sprinters result from the fact that muscle mass plays a significantly role in their efforts^13^, which was shown in the currently presented research in the form of a high level of sprinters’ mesomorphy. As previous studies have shown, effective sprinting requires strong deep muscles of the trunk (*psoas major, transversus abdominis, and multifidus muscle*), which, acting earlier than other muscles, are the basis of limb strength and thus affect the athlete’s sports results^30^. Also, Tottori et al.^31^, comparing the cross-sectional areas of the trunk and lower limb muscles in sprinters, found significantly larger *psoas major* and *gluteus maximus* muscles than non-runners. In addition, they not only correlated significantly with 100 m sprint performance, but they turned out to be good predictors of top performance over that distance.

The upper body and arms also play an important role in running, providing balance and promoting efficient movement. In our research, the transverse dimensions of the trunk (shoulder width, hip width), skeletal mass and limb circumferences were significantly larger in sprinters, which is confirmed in other studies^31,32^. No such differentiation was found between long- and middle-distance runners, confirming previous observations^33,34^. The shape of the torso contributes to the locomotor efficiency and energetics of running, influencing the respiratory mechanics and biomechanics of the limbs with its morphology^33^. Studies of variations in trunk morphology in the context of locomotor ability have shown a relationship between trunk shape and running performance^33^. According to these results, people with a narrower torso can achieve higher speeds^33^, which was confirmed by our research. In addition, as other studies have shown^34^ less thoracic kyphosis and a flattened chest positively affect the mechanics of breathing (chest mobility). On the other hand, increased lordosis has a beneficial effect on the biomechanics of the lower limbs, playing an important role in mitigating the effects of shocks transmitted through the human spine during dynamic activities such as running^34^. The width of the pelvis also affects the work of the psoas major muscle, affecting its rotation capacity and hip flexors^35^.

The length proportions do not significantly differ between the examined groups of athletes. Slightly higher values of the lower limb length index and upper limb length index characterize long-distance runners, which was confirmed in other studies^9,36^. In the presented work, it was shown that sprinters are characterized by slightly shorter lower limbs compared to other groups of athletes. Research by other authors shows that long legs are beneficial in sprinters, but only to the optimal level correlating with their height^37^. If the lower limbs are above this optimal length, they can generate problems producing the high stride frequency that is a prerequisite for good^38^. In the group of middle-distance runners, slightly longer lower limbs and their segments were demonstrated, as noted by other authors^39^. Researchers also note that the main difference between long- and short-distance runners is stride length, not running pace^40^. Studies have shown that shorter distance runs require longer strides^41,42^, which may be reflected in the proportions between the lower limb segments to some extent.

Bereket^43^ noted that the body’s size and proportions affect the energy of locomotion and the speed of movement. It was found that taller people with a wider pelvis, having a longer lower leg move at a much higher optimal walking speed at a lower energy cost, which was justified, among others, by the importance of the length of the distal section in heat dissipation^44^. The analysis of intergroup differences of proportions within the lower limb of runners at various distances carried out in the current study provided interesting results. The relationship between the proximal and distal segments of the lower limb is noteworthy. It was found that long-distance runners have a relatively long shin (relative to the thigh), suggesting that long-term intense exercise may promote proportions that favore more efficient heat loss in the lower limb. On the other hand, sprinters have a significantly shorter shin in relation to the length of the thigh (crural index) compared to other groups of competitors. Also, Tomita et al.^45^ showed that the ratio of tibia length to femur length significantly correlated with running performance in sprinters, suggesting that this particular morphological factor may play an important role in achieving better running performance in specialized 400 m sprinters.

Likewise, the relative slenderness of the thigh and lower leg are significant factors in running economy^46^*.* In the presented studies, a significantly more massive skeleton, assessed by the width of the epiphyses, is characteristic of long-distance runners, which can be justified by the influence of varied effort^47,48^. The musculature of the limbs is shaped differently. Significantly larger circumferences of limb segments were found in sprinters compared to other groups of runners. Korhonen et al.^49^ showed that muscle thickness was a strong predictor of the braking forces generated during sprinting. Similarly, other studies have found that sprinters with higher lean body mass in the lower limbs showed higher mean power in the Wingate test^50^. Current research has shown that long- and middle-distance runners are characterized by slimmer limb segments compared to sprinters, which has been confirmed in the literature^39^.

The diversified energy cost of running over particular distances affects muscle mass development and body fatness^32^. In addition, the amount of subcutaneous adipose tissue in different body regions may be of practical importance, as changes in subcutaneous adipose tissue distribution are also associated with changes in running performance^51^. This enables skinfolds to be used as useful predictors of running performance. As noted by other authors^52^, also in the presented study, it was found that little subcutaneous fat characterizes groups of short- and middle-distance runners. Thicker skinfolds are characteristic of long-distance runners, which may reflect differences in their metabolism^52,53^. Subcutaneous adipose tissue is an important and most natural reservoir of energy necessary for athletes to perform long-term efforts^54^. It also performs important endocrine functions^55^. The distribution of subcutaneous fat is slightly different in the studied groups of athletes. Limb fatness relative to the trunk is low in sprinters and middle-distance runners, which can be explained by muscle group-specific adipose tissue loss due to systematic training^52^.

Body composition is also an important characteristic of runners. Its basic components, adipose tissue and lean mass with cellular and acellular fractions, strongly correlate with the ability to increase muscle strength, contributing to improved performance and running economy^56^. Using anthropometric and DXA methods to analyze body composition, it was shown that the low cost of locomotor energy was associated only with parameters indicating relative slenderness of the body^46^. In a current BIA study, middle- and long-distance runners have been shown to have a slightly higher percentage of fat and extracellular mass compared to sprinters, reflecting their slimmer physique^27^. Extracellular mass is known to include connective tissues such as collagen, elastin, skin, tendons and bones^57,58^. In turn, the body cell mass responsible for metabolism is significantly higher in short-distance runners^13^. Also, the distribution of individual athletes’ points in the system of three variables of body composition assessed with the χ^2^ test showed statistically significant differences between groups. Studies by other authors confirm that the differences in the body composition of runners are a consequence of different workloads resulting from the length of the distance covered^18,59^.

The morphological characteristics of runners are supplemented by the somatotypological assessment, which allows for determining the size of endomorphy, mesomorphy and ectomorphy in the body structure^16^. It should be noted that the athletes are slender with moderate musculature and low body fat, which has also been noted in other studies. SANOVA showed a statistically significant difference in the somatotypes of the tested runners. Middle-distance runners are mesomorph-ectomorph (1.60–3.82–3.81), and sprinters (1.68–4.94–2.90) and long-distance runners (2.11–4.72–3.36) are an ectomorphic mesomorph. The somatotypes of the examined athletes are similar to the somatotypes of Croatian runners over various distances^27^ and participants of the Olympic Games in 1984^16^. Runners from Croatia presenting a higher sports level (were in the top 15 on the Croatian Athletic Association rank list for the specific event) than the academic runners surveyed in actual research are characterized by higher values of endomorphy and ectomorphy (S: 2.0–4.2–3.0; M: 2.1–3.8–3.3; L: 2.6–3.5–3.7), while the mesomorphy in the groups of short- and long-distance runners is slightly lower. In turn, the participants of the 1984 Olympics clearly dominate the size of the mesomorphic component (S: 1.7–5.2–2.8; 400 m runners 1.5–4.6–3.4; M: 1.5–4.3–3.6; L: 1.4–4.2–3.7), which is a consequence of their higher sports level.

Using principal components analysis made it possible to isolate three principal components explaining the problem in approximately 74%. The first principal component characterizes the overall size and development of musculature and does not significantly differ between the examined groups of runners. The second component differs significantly between short-distance and middle-distance competitors. It is positively correlated with muscle circumferences and negatively correlated with lower limb length, thigh length and lower leg length, confirming the observations of other authors indicating the relationship between a more linear body and running economy^46,60^. The third principal component characterizes the body’s subcutaneous fat in terms of distribution. As previous studies have shown, the anthropometric assessment of athletes should include the assessment of all skinfolds, as the reduction of subcutaneous fat is not the same in all segments. In the case of runners, it concerns mainly the lower limbs^52^.

The morphological distinctiveness of distance runners is expressed in overall body size, muscularity and fatness as well as lower limb proportions. These characteristics should be particularly controlled by coaches during the selection process for individual competitions, as well as throughout the training period. The optimization of selection processes and training methods based on these characteristics seems to be an important element for achieving success in running at different distances.

## Conclusions

The conducted analyses indicate the diversification of anthropometric profiles in runners of different distances. Sprinters dominate a more massive body shape, shorter lower legs in relation to the length of the thigh, broader shoulders and narrower hips, greater musculature and cellular mass. Long-distance runners are characterized by a slim figure, a high crural index, a slightly wider pelvis in relation to the width of the shoulders, and the greatest adiposity and extracellular mass. Middle-distance runners are the slimmest, and have a narrow trunk and little subcutaneous fat. The physique of sprinters and long-distance runners is dominated by mesomorphy, while middle-distance runners are mesomorph-ectomorph. The principal component analysis reduced the multidimensional structure to three variables: overall body size, limbs musculature and the length of the lower limb together with its segments, and body fatness. This approach emphasizes the morphological distinctiveness of runners at particular distances and allows the use of somatic features as predictors of running performance.

Our study revealed essential differences between the student athletes in the three running disciplines. However, in the future we plan to link this anthropometric diversity to the other relevant fields as biomechanics to find general success predictors in running. Moreover, we would like to confront our results with a similar study conducted on the cohort of the elite-level runners from the abovementioned disciplines. Therefore, we would be able to provide coaches and selectors with the robust set of success predictors both in the running performed on the elite and non-elite level. This way, future research of the morphological diversity of runners should focus on these somatic features indicated in this manuscript. The future study should be conducted in more homogeneous groups in terms of sports level, especially groups of high-level athletes and should analyze the importance of indicated features for better performance.

## Study limitations

The small size of the analyzed groups of athletes may limit the interpretation of the results. While the analysis was successful, increasing the sample size would have given a clearer picture of intergroup variability. In addition to that, the competitors were qualified to groups on the basis of a survey concerning the competitions and distances they competed on. However, this division may not be completed, as it happens that athletes change the distance during their career, which may affect the picture of morphological diversity. Interpretation may also be limited by the varied sports’ level of the respondents.

## Data Availability

The datasets used and/or analyzed during the current study are available from the corresponding author on reasonable request.
